# In Silico Methodologies to Improve Antioxidants’ Characterization from Marine Organisms

**DOI:** 10.3390/antiox12030710

**Published:** 2023-03-13

**Authors:** Chiara Lauritano, Eleonora Montuori, Gabriele De Falco, Sabrina Carrella

**Affiliations:** 1 Ecosustainable Marine Biotechnology Department, Stazione Zoologica Anton Dohrn, Via Acton 55, 80133 Napoli, Italy; 2 Department of Chemical, Biological, Pharmaceutical and Environmental Sciences, University of Messina, Viale F. Stagno d’Alcontres 31, 98166 Messina, Italy

**Keywords:** marine organisms, antioxidant compounds, enzymes, in silico search, bioinformatic analyses

## Abstract

Marine organisms have been reported to be valuable sources of bioactive molecules that have found applications in different industrial fields. From organism sampling to the identification and bioactivity characterization of a specific compound, different steps are necessary, which are time- and cost-consuming. Thanks to the advent of the -omic era, numerous genome, metagenome, transcriptome, metatranscriptome, proteome and microbiome data have been reported and deposited in public databases. These advancements have been fundamental for the development of in silico strategies for basic and applied research. In silico studies represent a convenient and efficient approach to the bioactivity prediction of known and newly identified marine molecules, reducing the time and costs of “wet-lab” experiments. This review focuses on in silico approaches applied to bioactive molecule discoveries from marine organisms. When available, validation studies reporting a bioactivity assay to confirm the presence of an antioxidant molecule or enzyme are reported, as well. Overall, this review suggests that in silico approaches can offer a valuable alternative to most expensive approaches and proposes them as a little explored field in which to invest.

## 1. Introduction

The Earth’s surface has been reported to be covered by the 70% water [1]. Marine habitats can be very variable, e.g., ranging from temperate to polar environments, from shallow to deep and from high salinity to low salinity [2,3]. In addition, natural acidified sites are available at sea, such as volcanic CO_2_ vents [4,5]. In concomitance with the natural environmental variables, other changes can alter the physiological homeostasis of a marine organism, including climate changes, pollutants and other perturbances derived by human activities that may induce a stress response [6,7,8,9].

The marine environment is characterized by huge biodiversity in terms of species as well as chemical diversity. According to the World Register of Marine Species (known as WORMS; https://www.marinespecies.org/; accessed on 11 January 2023) [10,11], there are currently 241,487 accepted marine species. Several studies and research projects are continually describing new species and, considering the less explored areas of the planet, such as extreme environments, this species number is expected to grow. Regarding their chemical diversity, marine organisms are known to produce a plethora of compounds belonging to the primary and secondary metabolism, sometimes with chemical structures unique to the marine environment. According to the database MarinLit, there are currently 39,365 articles on marine natural products research and 39,596 known compounds (https://marinlit.rsc.org/; accessed on 11 January 2023). These molecules can be physiologically produced in natural conditions or induced by an external stimulus. Harsh marine environmental conditions have favored the production of a great variety of compounds with unique structural and functional features, often with higher significant bioactivity compared to terrestrial molecules [12]. Compounds can be released as a defensive strategy against predators, to attract a partner, to avoid microbial infections, to communicate with other species, for cell–cell communication and for many other purposes [13,14,15].

**Figure 1:**
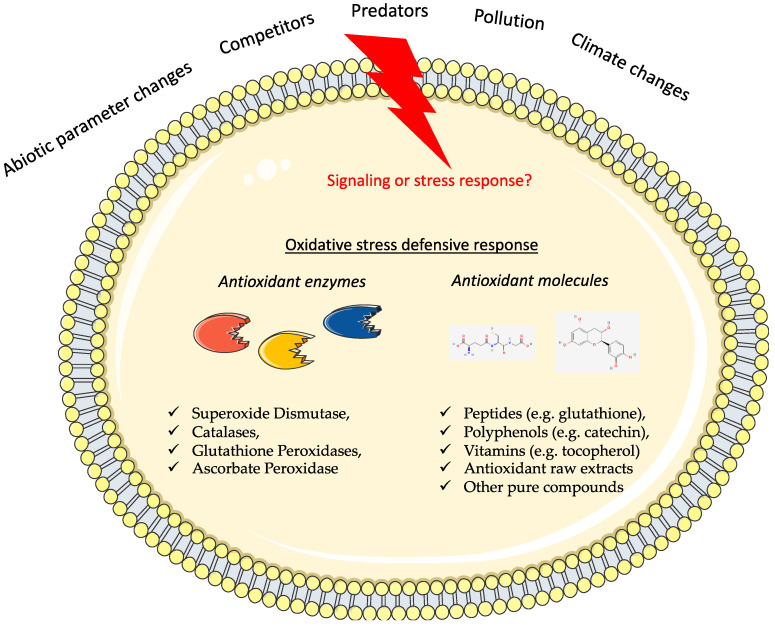
Schematic representation of enzymatic and non-enzymatic defense strategies, reporting some examples of oxidative stress inducers, as well as examples of antioxidant enzymes and molecules.

As a result of changes in environmental conditions, pollution, climate changes and interactions with other species, marine organisms are continuously exposed to oxidative stress [16,17]. Oxidative stress can be considered an imbalance between the generation of free radicals, such as reactive oxygen species (ROS), and the antioxidant defense system of an organism [18]. Examples of ROS are superoxide anion (O_2_^−^), hydrogen peroxide (H_2_O_2_) and hydroxyl radical (HO•) [19]. In general, ROS, at low concentrations, have been reported to have important roles in stress perception, pathogen recognition, programmed cell death and others [20], but when produced at high concentrations, are known to induce damages to nucleic acids, proteins, lipids and carbohydrates [17], inducing enzyme denaturation, lipid peroxidation and blockage of various cellular functions [21]. However, elevated ROS have been also reported as signaling compounds in order to maintain/regulate physiological activities [22]. How do marine organisms respond to ROS stress induction? Cells have the possibility of activating a series of defense strategies, which include both the activation of antioxidant enzymes and the production of antioxidant molecules, known as enzymatic and non-enzymatic defense mechanisms (Figure 1) [21].

Regarding the antioxidant enzymes, there are various examples involved in the detoxification of ROS, such as superoxide dismutases (SODs), catalases (CATs), glutathione peroxidases (GPXs) and ascorbate peroxidase (APX) [20]. SODs convert superoxide radicals into hydrogen peroxide (H_2_O_2_), which is further converted into water and divalent oxygen (O_2_) by CAT and GPX [23]. SOD1 has been reported to be mainly located in the cytosol, while SOD2 in the mitochondrial matrix. SODs avoid superoxide accumulation and have been more associated with stress than signaling [22]. However, it has been reported that superoxide is able to inactivate specific proteins, does not induce indiscriminate protein damages and is the major ROS-regulating autophagy [22]. On the contrary, hydroxyl radical has been reported to induce indiscriminate oxidization of biological molecules, thus causing damage and genomic instability [24]. APX also catalyzes the reduction of H_2_O_2_ to water by ascorbate and produces monodehydroascorbate and dehydroascorbate [25]. Ascorbate can be regenerated by monodehydroascorbate reductase and dehydroascorbate reductase by using NADH and reduced glutathione, respectively. Glutathione is synthetized by glutathione synthetase (GSH), which can be found in an oxidized or reduced state [23]. The oxidized state can be reconverted into the reduced state thanks to glutathione reductase (GR), which makes the thiol group of cysteinyl residue available as a source of one reducing equivalent. In addition to ROS quantities, their compartmentalization in mitochondria or cytosol is also known to dictate signaling/oxidative stress responses [22]. Antioxidants enzymes, such as SOD and CAT, have been found activated upon stress exposure in several marine organisms, from marine vertebrates and invertebrates to plants [5,26,27,28,29,30,31]. 

**Figure 2:**
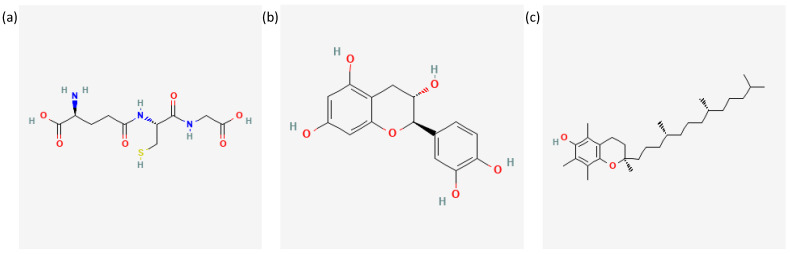
Antioxidant scavenger molecules: (**a**) glutathione [33], (**b**) catechin [34], (**c**) vitamin E [35]. Chemical structures are retrieved from the database PubChem (https://pubchem.ncbi.nlm.nih.gov/; accessed on 21 January 2023).

The non-enzymatic defense strategies include a series of scavenger molecules such as peptides (e.g., glutathione), polyphenols (around 8000 compounds known, e.g., catechin) and vitamins, such as tocopherol (vitamin E) (Figure 2) [32].

In addition to these compounds, marine organisms have been shown to produce several metabolites with antioxidant activity. Depending on the studied organisms, the chemical extraction protocols used and the sampling/culturing conditions, different intensities of antioxidant activity have been found (as reviewed by Vladkova and colleagues [21]). Antioxidant activities have been observed from marine fungi to vertebrates, collected from all over the globe. However, the compound/s responsible of the activity observed is still often unknown and further chemical analyses are necessary to clarify the chemical structure of the compound responsible for the raw extract antioxidant activity [21,36,37,38]. This research approach involves the sampling/culturing of a marine organism, the preparation of chemical extracts/fractions, bioactivity testing and identification of the molecular structure of the bioactive compound/s.

The aim of the current review is to summarize recent studies that used an in silico approach to identify and characterize antioxidant compounds. Bioinformatic tools have allowed researchers to identify antioxidants from marine organisms by analyzing available sequences in public databases, as well as ascertain new details concerning antioxidants by using docking tools on specific molecules and/or studying possible interactions between a marine compound and a specific target, such as a cellular receptor.

### What Is Already Available on the Market?

There are many synthetic antioxidants on the market, used in the cosmeceutical and pharmaceutical industries, such as butylated hydroxytoluene (BHT), butylated hydroxyanisole (BHA), tert-butylhydroquinone (TBHQ) and propyl gallate (PG), which have been used to retard the oxidation and peroxidation processes. The use of synthetic antioxidants must be under strict regulation due to potential health hazards [39,40]. Therefore, researchers are increasingly looking for natural sources from which to obtain antioxidant compounds, encouraged by the huge demand for these compounds mainly in the nutraceutical, cosmeceutical and pharmaceutical sectors. There is a considerable interest in the development of antioxidants from natural sources, including marine flora and fauna [41]. The antioxidant activity of marine species has been widely studied [42,43]. In addition, recently, many researchers have also explored the potential applications of protein hydrolysates from by-products of fish processing as alternative sources of antioxidants in light of the circular economy and taking an eco-friendly and eco-sustainable approach to the discovery of new or improved products/molecules. To date, marine resources are still little explored considering the vast number of species that populate the sea. Many studies suggest marine organisms as a great source of natural products with applications in various industrial fields [44,45]. For example, collagen is already used for skincare products to prevent and repair the damage caused by physiological or environmental events. Based on the safety of marine collagen [46], researchers are exploring its isolation from marine species. In particular, fish, jellyfish and sponge, as well as fish discards, may be excellent sources of collagen for uses in pharmaceutical and cosmeceutical preparations [47,48,49,50]. These new sources can also offer valuable alternatives to porcine- and bovine-derived products, which are forbidden by religious constraints for many people [49].

There are already many examples of marine-derived products with antioxidant properties that are used, above all in the cosmetic and nutraceutical industries. For example, marine microalgal extracts are rich in cosmeceutical ingredients such as phlorotannins, polysaccharides, carotenoid pigments (fucoxanthin) and fucosterol [51,52,53]. Marine microalgae may deliver antioxidant metabolites in the forms of biomass, crude total extracts, fractions and pure substances. The microalga *Chlorella* sp., in particular, contains various valuable proteins that have been reported to have great potential for use in various cosmetic products [41]. DERMOCHLORELLA DP (by CODIF) is an example of a product based on an extract of *Chlorella vulgaris*, rich in peptides and amino acids, which was found to stimulate the synthesis of collagen in the skin, thus promoting tissue regeneration and the reduction of wrinkles (https://www.codif-tn.com/en/; accessed on 17 January 2023). The company Biosearch (https://www.biosearchsrl.com/products/; accessed on 5 January 2023) produces Skinrep, a water extract derived from the marine green microalgae *Tetraselmis suecica*, which has antioxidant, anti-inflammatory and repairing activity in human cell lines and epidermal tissue. The microalga *Tetraselmis suecica* reduces oxidative stress and induces repair mechanisms in human cells [54]. Skinrep is now used for the preparation of three products: a daily anti-aging cream Anti-Age UV Shield, a new anti-pollution cosmetic called Urban Serum and a daily lip treatment called Bioactive Lip Care. 

Other antioxidant compounds produced in high quantities by microalgae, which have been receiving increasing attention during the last decades, are carotenoids. Carotenoids are accessory pigments that play the primary role in light harvesting and are reported to have a potential function in preventing adverse health conditions in humans. They are a class of isoprenoids, comprising β-carotene, lycopene, astaxanthin and lutein [55], and play a protective role by preventing the formation of reactive oxygen species [56,57]. According to Markets and Markets (https://www.marketsandmarkets.com/Market-Reports/carotenoid-market-158421566.html; accessed on 17 January 2023), the carotenoids market is expected to grow from USD 1.5 billion in 2019 to USD 2.0 billion by 2026, with a compound annual growth rate (CAGR) of 4.2%. Carotenoids find applications in several sectors. Among them is food preservation. Carotenoids, thanks to their antioxidant properties, are able to neutralize free radicals and reactive oxygen species [58] and are reported to slowing the aging process [59]. Carotenoids find applications in cosmetics, as well, as they protect the skin from UV, meaning they are often used in tanning lotions and protective creams. Examples of cosmetics based on carotenoids extracted from marine organisms come from Eclae (https://www.eclae.com/; accessed on 13 January 2023), which sells Mousse Exquise based on extracts of *Dunaliella salina* [60] with antioxidant properties for deep cleansing of the face without irritating the skin and to soften the skin thanks to its airy texture.

Extracts from marine algae such as *Undaria pinnatifida*, *Polysiphonia lanosa* and *Durvillaea antarctica* have been used in skin protection, as well [51,52,53]. Codif Technologie Naturelle (https://www.codif-tn.com/en/; accessed on 17 January 2023), in 2020, launched its “Short Range of Cosmetic Food Ingredients from the Sea” project on the market, including Matrigenics 14G (based on extracts of the brown macroalga *Undaria pinnatifida*), which is rich in wakamic ester and found to reactivate dormant genes to restructure the extra-cellular matrix; WAKAPAMP LIPS, based on extracts obtained by supercritical CO_2_ extraction from *U. pinnatifida*, reported to provide lips with the benefits of anti-aging, restructuring and plumping care; and the detox EARTH MARINE WATER, which is water of marine origin, rich in minerals, applied to re-mineralize, detoxify and strengthen the skin. OSEA (https://oseamalibu.com/; accessed on 21 January 2023) marketed a series of products with antioxidant, anti-aging, anti-wrinkle properties based on marine extracts from *Undaria pinnatifica,* such as “Undaria Algae Body Oil” and “Undaria Algae body polish” (https://oseamalibu.com/search?q=undaria&options%5Bprefix%5D=last; accessed on 21 January 2023). OSEA also produces a ‘’Blemish Balm’’ based on extracts of *Macrocystis pyrifera* reported to calm and clarify the skin and deliver balanced hydration (https://oseamalibu.com/products/blemish-balm; accessed on 21 January 2023). ASPAR’AGE™ (https://www.seppic.com/en/wesource/asparage; accessed on 21 January 2023), an extract of *Asparagopsis armata*, is incorporated in some lotions with anti-aging properties as it contains mycosporine-like amino acids, found in marine algae, which are antioxidant and skin protective [61,62]. 

Regarding nutritional applications, the microalgal *Schizochytium* sp. oil was obtained and authorized by the United States to be used as a novel food ingredient [63] due to its high content of DHA (n-3), squalene and phytosterols, and since it also contains three times less cholesterol than fish oil [64,65]. The *Schizochytium* oil is produced according to the Food Safety System Certification (FSSC) 22000, which includes hazard analysis critical control point (HACCP) principles [63]. On 18 July 2019, the company Bioplus Life Sciences submitted a request to the European Commission to authorize the use of *Schizochytrium* oil, obtained from *Schizochytrium* sp. ATCC 20889, in infant formulae food categories [66] (https://efsa.onlinelibrary.wiley.com/doi/full/10.2903/j.efsa.2020.6242; accessed on 21 January 2023). Edible seaweeds are also an excellent source of health-beneficial substances such as dietary fiber, essential amino acids, vitamins, phytochemicals, polyunsaturated fatty acids and minerals [67], and they show antioxidant properties. The main genera grown for nutritional purposes are *Laminaria*, *Undaria* and *Porphyra* [64]. Bioalghe (https://www.bioalghe.it/; consulted on 19 January 2023) marketed a series of products based on seaweed and microalgae. Among them, there is the food supplement ConsonniBioalghe, based on seaweed extracts of *Porphyra umbilicalis* with omega 3 EPA and DHA, which contribute to the proper functioning of the heart and brain and to the maintenance of normal levels of cholesterol and triglycerides (https://www.bioalghe.it/porphyra-umbilicalis; accessed on 19 January 2023). Phenolic compounds found in marine algae are known for their activity against oxidative stress [68], with phlorotannins (most abundant in brown algae [69]) used like active ingredients in nutraceutical compositions [70].

Chitosamin^®^ is a dietary supplement with an antioxidant effect. It is essentially chitosan with a high molecular weight ~100 kDa (derived from marine outer skeleton), which is used as a preparation in the antioxidant treatment of various diseases, including renal failure [71]. Chitosamin^®^ was shown to induce a decrease in lipid hydroperoxides and uremic toxins in the gastrointestinal tract, inhibiting oxidative stress in the human systemic circulation [72]. A similar product based on chitosan, named Longlife chitosan, is produced by LongLife (https://www.slowfarma.com/longlife-chitosan-84-tavolette.html; accessed on 23 January 2023). The seafood processing industry can offer a possible source of chitin and chitosan, allowing for a circular economy of marine resources [71]. Examples of industry waste that may be used for this purpose are shells, scales, tails, heads and guts. 

Many marine-derived proteins are used as food components [69]. Some examples are collagen [73,74], gelatin [75] and albumin [76]. These protein are enzymatically hydrolyzed to produce bioactive peptides that can be used as nutraceuticals and also for their antioxidant properties [77,78,79,80,81,82]. Albumin, in particular, extracted form mollusks, crustaceans and low-fat fish, finds uses in the food industry for whipping, suspending and as a stabilizing agent thanks to its anticoagulant and antioxidant properties [76].

Several marine organisms are also well known as sources of omega-3 fatty acids [83], meaning they find applications in nutraceuticals (e.g., fish oil), fortification of livestock, feed and infant formula. Omega-3 had numerous health benefits including strong antioxidant properties [69,83]. 

## 2. Overview of Tools for In Silico Prediction of Bioactive Peptides

Prediction methods have become more and more helpful in order to elucidate the structure, function and properties of macromolecules or for rational drug design. In silico identification is also used to find and characterize putative ligands of proteins. This is important not only for drug discovery and design but also to specify protein–protein interaction networks by investigating putative protein binding sites and revealing the number and type of protein interaction partners [84]. In this section, we analyze different tools for in silico prediction: docking, bioactivity of peptides, protein structure and pharmacophore modeling. 

### 2.1. Docking Prediction Tools

Molecular docking is a method that characterizes how two or more macromolecules, such as proteins, enzymes and drugs, interact [85]. It is used to predict the affinity strength of the molecules and their binding mode, then obtain a structure-based drug design [86]. Molecular docking can occur in three different ways, depending on the conformation of the substrates and receptors: rigid, flexible and semi-flexible. In the first, the conformation of the ligands and receptors is assumed to be rigid, meaning their shape does not change during the process, only the position and the orientation of the molecules [87]. This method fits for the identification of interactions between big molecules, i.e., protein–protein, protein–nucleic acid. Flexible docking is applied to evaluate ligands and receptors with a conformation that changes liberally since this kind of calculation is similar to the real docking conditions and has high accuracy. However, it is computationally intensive and time-consuming [86]. Semi-flexible docking is an evaluation where the conformation of the ligands is permitted to change within a certain threshold, whereas the receptors are fixed. This method has been used to identify the interactions between small molecules and macromolecules [88]. 

One of the tools used to predict molecular docking is AutoDock4 (https://autodock.scripps.edu/; accessed on 21 January 2023) (Table 1). 

The software predicts bound conformations and binding energies of ligands with macromolecules targets [109]. To do so, it uses a grid-based method, where the target protein is placed inside a grid, then a probe atom is placed at each grid point and the interaction energy between the two is computed, with the value stored in the grid [89]. Ultimately, the grid with the energetic values is used as a reference during a flexible docking experiment. Alternatively, AutoDock Vina (https://vina.scripps.edu/; accessed on 21 January 2023) (Table 1) is a new program for the prediction of flexible docking. It is an improvement of AutoDock4 with greater accuracy of the binding mode predictions and a two orders of magnitude speed-up thanks to using multithreading [90]. 

Another tool to predict molecular docking is Flexible CDOCKER (Table 1), which is an extension of CHARMm (https://charmm.chemistry.harvard.edu/main.php; accessed on 22 February 2023). This method allows researchers to study molecular docking both by evaluating atomically detailed side chain flexibility and by using grid-based methods, as well as explore the conformational space simultaneously of ligands and protein configurations [91]. This is the main difference between CDOCKER and AutoDock, which samples the protein and ligand space independently.

Beyond those, another useful tool is FireDock (http://bioinfo3d.cs.tau.ac.il/FireDock; accessed on 21 January 2023) (Table 1), which offers a method for the rescoring and refinement of docking solutions. This tool improves the binding by allowing flexibility in the side chains, and during rigid body optimization and scoring phases, the atomic radii of the molecules are smoothed [92]. The scoring stages are based on Atomic Contact Energy (ACE), van der Waals interactions and electrostatic and binding free energy evaluations.

In addition, PatchDock (https://bioinfo3d.cs.tau.ac.il/PatchDock; accessed on 21 January 2023) (Table 1) is a tool developed by the same group of FireDock, which predicts protein–protein and protein–small molecule interactions. This tool categorizes the Connolly dot surface representation of the molecules into concave, convex and flat patches. The Connolly dot surface describes the boundary of a molecular structure to its environment [110]. Subsequentially, the patches are matched by complementarity in order to generate candidate transformations, and each of them is further analyzed in a scoring stage based on geometric fit and atomic desolvation energy [93].

Moreover, in order to improve cross-platform compatibility and assessment metrics for analyzing compound candidates, a flexible and easy-to-use graphical interface called Dockey (downloadable at https://github.com/lmdu/dockey accessed on 21 February 2023) has been developed (Table 1) [94]. Specifically, small molecules’ and proteins’ non-covalent interactions are analyzed by this tool and cross-docking between multiple receptors and ligands is performed. Compared to other tools, it can automatically dock more ligands to different receptors, and it can detect the corresponding docking solutions in parallel [94].

Furthermore, iMOLSDOCK (downloadable at https://sourceforge.net/projects/mols2-0/files/; accessed on 21 February 2023) (Table 1), an extension of the Mutually Orthogonal Latin Squares (MOLS), is an induced-fit docking algorithm that has made calculation faster due to code optimization in the docking tool and an improved scoring system. It also allows residues to deviate from the initial position due to increased receptor flexibility [95].

### 2.2. Bioactive Compound Prediction Tools

Bioactive peptides, like some antioxidants, have a precursor stage and only become mature and active after being processed by enzymatic hydrolysis [111]. This property makes the screening of a specific bioactive peptide more complex. However, in silico prediction tools helped overcome this challenge by allowing researchers to select the suitable protein and enzyme combination that could potentially generate the specific peptide [112]. 

BIOPEP-UWM (https://biochemia.uwm.edu.pl/; accessed on 21 January 2023) (Table 1) is one of the tools used for the prediction of bioactive peptides. Formerly known as BIOPEP, it is a database of sensory peptides and amino acids, that includes: information about taste and structure written with chemical codes (such as SMILES), sequences written in one-letter code, bioactivity information (such as inhibition of proteolytic enzymes) and ID numbers from other databases [96]. In addition, it contains some tools for the determination of precursors that may contain a specific peptide, simulating proteolysis. In recent years, information had been added, updated and completed on proteins, allergenic proteins and their epitopes, sensory peptides and amino acids [97]. In addition, there is a new function that allows the database to be expanded through the submission of new peptides from users.

Beyond that, there is Peptide Ranker (PepRank) (http://distilldeep.ucd.ie/PeptideRanker/; accessed on 21 February 2023) (Table 1), which predicts whether a peptide is bioactive. For a list of peptides, PepRank evaluates the probabilities to find bioactive ones and rank them from 0 to 1 [98]. Thus, the listed peptides are considered active based on the selected threshold, generally referred to as 0.5 [113]. To complete its task, the tool considers both the amino acid organization and the effects of extracellular status on predictions [114]. 

Additionally, for evaluation of the allergenicity of a molecule, there is the tool AllergenFP (http://ddg-pharmfac.net/AllergenFP/; accessed on 21 February 2023) (Table 1). It is an alignment-free descriptor-based fingerprint approach that has a four-step algorithm: (1) the protein sequences are categorized by amino acid properties (such as size, secondary structure motif-forming propensities); (2) the formed strings are converted into vectors of equal length; (3) the vectors are converted into binary fingerprints; (4) they are compared in terms of the Tanimoto coefficient [99,107].

Furthermore, ToxinPred (https://webs.iiitd.edu.in/raghava/toxinpred/index.html; accessed on 22 February 2023) (Table 1) is a tool that helps to identify the toxicity of peptides and toxic regions in proteins. It also has a module in which it is possible to design peptides with single mutations and then predict the toxicity of the mutants [115]. This tool is based on a hybrid model, combining a dipeptide-based model with information about various motifs from toxic peptides [115].

### 2.3. Protein Structure Prediction Tools

In silico prediction of the structure and functional sites of proteins provides useful information before experiments for the design of drugs or inhibitors and antagonists, or to introduce targeted mutations that modify the protein functions [84]. Furthermore, it can help in modeling the three-dimensional structure of protein–ligand complexes [116]. 

Firstly, ExPASy (https://www.expasy.org/; accessed on 21 January 2023) (Table 1) is a database that comprehends resources from the Swiss Institute of Bioinformatics (SIB) dedicated to proteomics (such as post-translational modifications, protein identification), genomics, structural bioinformatics, phylogeny, population genetics, transcriptomics, imaging, systems biology, biophysics, population genetics and drug design [101]. In addition, also from SIB, is the SWISS-MODEL Repository (https://swissmodel.expasy.org/repository; accessed on 21 January 2023) (Table 1), a database of 3D structure proteins. Specifically, it generates models based on a fully automated homology modeling approach: select a suitable template, align target sequences with the template, build a model, minimize and refine energy and assess the quality of the model [102]. 

Moreover, as a tool for prediction of 3D protein structures, there is I-TASSER (https://zhanggroup.org/I-TASSER/; accessed on 21 January 2023) (Table 1). Firstly, there is a threading stage, where there is the identification of template proteins from databases via bioinformatics procedures; then, there is a structural assembly stage, where continuous sequences in the threading alignments are removed from the template and used to assemble structural conformations; in the end, there are the model selection and refinement stage, where the assembly simulation is performed again, and the structure-based functional annotation stage, where the function of the protein is assessed based on structural similarities to other known 3D protein models [103].

Furthermore, in order to predict protein residue-level annotation, protein function and chemically modified peptides’ function, the tool Pfeature has been developed (https://webs.iiitd.edu.in/raghava/pfeature/; accessed on 22 February 2023) (Table 1). It is divided into six categories: “composition” to compute the majority of the compositional features; “binary profiles” to compute the composition and position of each type of residues; “evolutionary information” to compute information about protein evolution using a position-specific scoring matrix based on PSI-BLAST; “structural features” to compute structural characteristics and descriptors from the tertiary structure of a protein; “patterns” to compute pattern-based descriptors; and “model building” to develop classification and regression models [104].

Furthermore, AlphaFold (https://alphafold.ebi.ac.uk/; accessed on 22 February 2023) (Table 1) is a novel machine learning approach that uses physical and biological information about protein structure, exploiting multi-sequence alignments, to design a deep learning algorithm [105]. The strength of this tool resides in the prediction of the protein structure with atomic accuracy even when there are no similar structures known.

### 2.4. Pharmacophore Modeling Tools

Pharmacophore modeling is based on the hypothesis that having shared chemical properties and functions, and also keeping a similar geometric conformation, equates to have activity on the same target [117]. Generally, pharmacophore modeling can be performed by following two different approaches: “structure-based” and “ligand-based”. The first identifies potential drugs starting from information on the targets (such as receptors or enzymes). The latter uses the physiochemical properties of known ligands for drug development.

One of the tools used for pharmacophore modeling is Phase (https://www.schrodinger.com/products/phase; accessed on 23 February 2023) (Table 1). This tool uses a tree-based partitioning algorithm in order to identify the geometric arrangements of functional groups shared and essential to the activity of a set of ligands. Then, there is the validation of the pharmacophore hypothesis based on the rationalization of the binding affinities of training molecules of different activities, prediction of affinities of test molecules and retrieval of known activities from a database of drug-like molecules [106].

Furthermore, there is the tool MOE (https://www.chemcomp.com/Products.htm; accessed on 23 February 2023) (Table 1) [107], for which 3D pharmacophore queries may contain locations of properties or chemical groups or restrictions on shape. In MOE, the volume shape and location are defined by a single sphere or by merging many [118]. Additionally, several aligned molecules can be used as a consensus query in the 3D pharmacophore database. 

Furthermore, LigandScout (http://www.inteligand.com/ligandscout/; accessed on 23 February 2023) (Table 1) is a structure-based pharmacophore modeling tool. It includes a variety of chemical properties such as hydrogen bonding vectors, aromatic plane interactions and chargeable groups, and the advanced alignment algorithm also allows researchers to compare the common binding modes of pharmacophores and molecules [108].

## 3. In Silico Analysis and Validation to Discover Antioxidant Properties of Marine Origins 

In silico studies represent a convenient and efficient alternative for the prediction of novel bioactivities of known and newly identified molecules. Marine natural compounds and bioproducts represent an invaluable source of bioactive molecules potentially able to act on multiple related targets due to their versatile scaffolds and functional groups. Here, we describe how the use of in silico prediction tools could be helpful in the prediction of possible mechanisms of action by which the marine molecules could exert their antioxidant activities. The main tools used in these studies rely on docking analysis or prediction of peptide and protein structures and activities. It is important to highlight that these in silico strategies could improve the research approaches for antioxidant discovery by reducing the time and costs of “wet-lab” experiments. Additionally, they could also be used as tools to develop the main “dry-lab” hypotheses, which can be further validated through focused “wet-lab” experiments. Here, we report some examples of the use of various tools applied to make marine bioactivity discoveries.

### 3.1. Molecular Docking Prediction and Validation

Marine algae have been found have excellent nutritional and potential therapeutic properties. Due to the numerous health benefits, food rich in antioxidants has become an essential part of the human diet. The presence of phenols and flavonoids in natural food can be associated with antioxidant capability. *Caulerpa racemosa*, a green macroalgae, is rich in phenolic compounds and widely consumed by humans. The fractionated polyphenolic extract of *Caulerpa racemosa* has been studied to explore its antidiabetic and anticancer potential [119]. An isolated compound, caulerpin, was used for in silico analysis. Caulerpin, belonging to a bisindole alkaloid group, was subjected to docking studies against α-glucosidase and estrogen receptor (ER) using the AutoDock Tools suite. The binding affinities to α-glucosidase and ER, respectively, indicate that the crude polyphenolic extract (CPE) of *C. racemosa* could potentially assist medicinal chemists in designing inhibitors against alpha-glucosidase enzymes in the human intestine to overcome diabetic conditions, and possibly developing the CPE as an anticancer drug lead for breast cancer thanks to its interaction with ER. Moreover, CPE of *C. racemosa* and its fractions (n-hexane, ethyl acetate, chloroform and distilled water) were tested for their total phenol and flavonoid contents and antioxidant potential. The antioxidant potential was evaluated using DPPH (2, 2-diphenyl-1-picryl-hydrazylhydrate) radical photometric assay, identifying a dose-dependent antioxidant activity. 

Similarly, DPPH assay was used to test the hexane ethyl acetate (HPAEtOAcE) fraction of *Pseudomonas aeruginosa* [120]. *P*. *aeruginosa* is an opportunistic pathogen and causes frequent infections in clinical settings; interestingly, the halophilic habitat of this organism has been shown to enable it to survive in high salt concentrations for a long time, with several genes showing adaptation to saline environments. The authors showed that the HPAEtOAcE fraction contained a new compound, which was identified as 5-(1H-indol-3-yl)-4-pentyl-1,3-oxazole-2-carboxylic acid (Compound **1**). The novelty of this compound was confirmed by SciFinder. The DPPH scavenging activity of HPAEtOAcE revealed substantial antioxidant activity compared to ascorbic acid (a standard antioxidant compound). This showed that HPAEtOAcE had effective antioxidant activity, and therefore, could represent a potential natural agent that could be applied in pharmaceutical applications similar to ascorbic acid. Interestingly, the novel Compound **1** structure identified in the HPAEtOAcE fraction was used for in silico studies using CDOCKER. Compound **1** showed good binding affinity at the active site of pantothenate synthetase (PS) of methicillin-resistant Staphylococcus aureus (MRSA), and the CDOCKER energy indicated high interaction, which can be explained by the formation of three H-bonds, five alkyls and one Pi–sulfur interaction with respective amino acids in PS. In particular, the main functional COOH group of Compound **1** formed two H-bonds with Arg 188 and Lys 150, respectively. In addition, the aliphatic and aromatic groups in Compound **1** formed van der Waals interactions with most of the active sites of the amino acids. These findings indicate that the novel 5-(1H-indol-3-yl)-4-pentyl-1,3-oxazole-2-carboxylic acid (Compound **1**) could be used as an effective antibiotic against MRSA. Furthermore, it could be tested in the future as an effective drug with high orientation against all pathogenic microbes. 

A different validation approach was used for the identification of the interaction between (−)-Muqubilin (Muq), a cyclic peroxide norterpene from a marine sponge, and the nuclear receptors (NRs) RARα, RXRα, PPARα and PPARγ [121]. The authors focused their attention on the identification of multiligand agents from natural sources potentially able to bind and activate nuclear receptors such as RXRα, RARα and PPARα/γ, which may have therapeutic potential for the treatment of neurological diseases such as Alzheimer’s disease thanks to their anti-inflammatory and antioxidant capacity. A ligand-based Virtual screening (VS) of the StOrMoDB database (https://stormodb.na.icb.cnr.it/stormodb; accessed on 21 January 2023) was performed using the featured SMARTS pattern querying option on database compounds (350 molecules of marine origin), to identify possible RXRα, RARα and PPARα/γ multiligands. For recent virtual screening reviews, please see Giordano et al. [117] and Zhu et al. [122]. The norterpene cyclic peroxide Muq proved to be a positive hit. Muq was then subjected to molecular docking and molecular dynamics (MD), to assess the propensity of the molecule to effectively bind the above-mentioned molecular targets. Docking studies were performed with AutoDock Vina1.1.2 [90] by using the crystallographic structures of RXRα, RARα, PPARα and PPARγ. The AutoDock Tools (ADT) package was used for both proteins and ligands to merge non-polar hydrogens, calculate Gasteiger charges and select the rotatable side-chain bonds. The binding modes found for Muq in the ligand-binding domain (LBD) of RXRα, PPARα and PPARγ were compatible with a full agonism since both the pattern of the polar interactions and the orientation of the hydrophobic tail recapitulated the binding mode of canonical agonists. Validation of the binding and the activity as an agonist was demonstrated using an in vitro luciferase assay. In this assay, the authors used reporters constituting hRXRα-LBD-Gal4, hPPARα-LBD-Gal4 and hPPARγ-LBD-Gal4 constructs transfected in COS-7 cells together with a TK-MH100x4-Luc containing the UAS enhancer elements. The ability to activate luciferase expression indicated that Muq is able to act as a full agonist for both hPPARα and hPPARγ receptors. However, the use of the construct reporter for RARα and hRARα-LBD-Gal4 fusion proteins showed that Muq was only a weak agonist for hRARα compared to RA in the reporter assay. Since MD predicted stable binding for the Muq–RARα complex, the ability of Muq to modulate the effect of retinoic acid (RA) was also evaluated. In vitro and in vivo experiments using zebrafish transgenic line Tg(12XRARE-elf1a:EGFP)sk72 highlighted that the co-administration of Muq and RA showed a strong additive effect, revealing an effect of Muq as a positive allosteric modulator for RARα.

Similarly, a very recent work [123] used AutoDock Vina 4.2.6 to study microalgal phytohormones’ (PHs’) ability to possibly bind to proteins involved in key cellular functions related to human metabolism and health protection/disease. The authors selected 53 naturally occurring PHs in order to cover large PH chemical diversity, including 22 cytokinins (CKs), 11 auxins (AUXs), 6 gibberellins (GAs), 6 strigolactones (SLs), 4 jasmonates (JAs), 2 brassinosteroids (BRs), abscisic acid (ABA) and salicylic acid (SA). A target fishing approach was used to predict the possible interactions between the selected PHs and proteins involved in human metabolism, cell growth or division and immune system functioning by using the ACFIS 2.0 online server (http://chemyang.ccnu.edu.cn/ccb/server/ACFIS2/#/home/index, accessed on 24 February 2023) [124]. Among the retrieved human targets mainly involved in the immune response, oxidative stress or cell cycle progression, most of them interacted with many PHs presenting redundant predicted binding affinities. The interactions between PHs and human proteins resulting as non-redundant, i.e., specific within a PH class, were further investigated in a second in silico step of analysis using AutoDock Vina 4.2.6, as mentioned before. This molecular docking analysis was performed with the aim to deeply investigate the ligand–receptor interactions of five selected cases. Interestingly, among the specific PH–human protein interactions that underwent docking analysis, two cytokinins, cis-zeatin (cZ) and N6-(D2-isopentenyl)-adenine (iP), were predicted to bind the cyclophilin peptidyl-prolyl cis-trans isomerase B (PPIB) protein [123]. In general, cyclophilins play a role in several intracellular processes such as oxidative stress, mitochondrial dysfunction, cell migration and apoptosis, with consequences for the development of cardiovascular diseases, neurodegeneration, cancer or viral infections. This approach allowed researchers to screen 53 PH compounds, highlighting PH–human protein interactions, representing a work that could pave the way to select compounds of potential interest to explore their in vitro and/or in vivo bioactivity. Further studies are needed to validate these interactions and PH bioactivity, enriching the interest in microalgae and their products as a resource for marketable products with human health benefits.

Additionally, the docking tools could be used as reverse docking tools for drug repositioning. Several studies have demonstrated that marine protein hydrolysates are abundant natural sources of antioxidants. Literature data highlight that tuna peptides have various functions. Tuna belongs to the Osteichthyes, Perciformes and Scombridae classes and is a highly migratory fish, well appreciated worldwide because of its high nutritional value and potential health benefits [125]. However, large quantities of fish protein are regarded as by-products and are discarded without any attempt at recovery or valorization. Tuna backbone isolated peptides have strong antioxidant activity [40], and peptides obtained from tuna cooking juice have angiotensin I-converting enzyme (ACE) inhibition activity that can be used to lower blood pressure [126]. Dark muscle contains abundant protein and is a promising and potential alternative protein source for the preparation of peptides. The peptide component of hydrolysate derived from the tuna dark muscle was sequenced via MALDI-TOF/TOF-MS. The peptides that were found in the highest proportions, KEFT (Lys-Glu-Phe-Thr), EEASA (Glu-Glu-Ala-Ser-Ala) and RYDD (Arg-Tyr-Asp-Asp), were studied via Discovery Studio 2016 software (DS 2016) to predict their functions [127]. DS 2016 is a new generation of molecular modeling software that is used in protein structure and drug discovery [128]. The analysis of the reverse docking, a method that does the opposite of virtual screening using docking [129], indicated that KEFT, EEASA and RYDD can bind to the Kelch-like ECH-associated protein 1 (Keap1), an important regulator of cellular oxidative stress via negative regulation of Nrf2/ARE antioxidant pathway. The molecular docking results were based on the receptor active site’s receptor–ligand–CDOCKER interaction energy (CIE). The number of interacting amino acid residues and other information to determine the extent of the peptides’ (KEFT, EEASA and RYDD) binding were compared to the TX6 binding characteristics, previously reported to bind Keap1 [130]. Interestingly, TX6 and the peptides KEFT, EEASA and RYDD have similar docking sites that are located at the same active site. In vivo validation, in mice treated with tuna dark muscle hydrolysates, highlighted that the antioxidant enzymes glutathione peroxidase (GSH-Px) and superoxide dismutase (SOD) increase and malondialdehyde (MDA) decreases in blood serum and liver with respect to saline-treated mice. Moreover, the antioxidant effect was analyzed at the mRNA-level by RT-qPCR analysis of Keap1 and the g2 regulatory subunit of AMP-activated protein kinase (Prkag2), which acts against oxidative damage. The RT-qPCR analysis revealed a decreased Keap1 transcript level and increased Prkag2 transcript level in the liver and brain of treated mice. Altogether, these data highlighted that the tuna dark muscle peptides could interact with Keap1 protein, inhibiting its function and activating the Nrf2/ARE antioxidant pathway, and at the same time, could increase the transcript level of Prkag2, involved in the AMPK pathway response against oxidative damage, indicating that the antioxidant activity of hydrolysates can be achieved by affecting multiple pathways [127]. 

All the mentioned docking tools could also be used for drug repositioning purposes. Drug repositioning is an “universal strategy” that involves the investigation of existing drugs with known activities for new therapeutic purposes. The use of in silico tools for drug repositioning makes it a time- and cost-effective strategy. One example is the study of the Cyclo (L-Leucyl-L-Prolyl) peptide/CLP, a natural marine metabolite that is well-recognized as an antimicrobial and antioxidant agent, identified to have antitumor activity via in silico prediction. The use of the PatchDock server (https://bio.tools/patchdock; accessed on 21 January 2023) allowed for the identification of the interaction between CLP and CD151 (Cluster of Differentiation 151), a member of the tetraspanin superfamily associated with malignancy of several human cancers [131]. The 3D structures of CD151 and CLP were submitted with the protein–ligand parameter in PatchDock, and the score of the docked complex was computed based on the principle of surface patch, molecular shape matching, filtering and scoring. Interestingly, protein–protein interaction of CD151 and epidermal growth factor receptor (EGFR) was performed using the FireDock server, which allows for flexible refinement and scoring of protein–protein docking solutions. Immunoprecipitation (IP) assay confirmed the interaction between EGFR and CD151 and that the presence of CLP reduces this interaction in triple negative breast cancer (TNBC) cell lines. These findings were further supported by the effect of CLP on the attenuation of cell cycle suppression and, in turn, growth and migration of TNCB cell lines, indicating that co-targeting of CD151 together with EGFR may be therapeutically beneficial against TNBC [131]. 

Other antioxidant molecules that underwent in silico drug repositioning were the marine carotenoids fucoxanthin (FX) and siphonaxanthin (SX), powerful antioxidants that present a variety of health benefits and industry applications. Docking calculations were carried out using AutoDock Vina (version 1.1.2) to identify the potential binding sites for the FX and SX models with the SARS-CoV-2 spike glycoprotein. In particular, potential binding was found of SX with the region corresponding to the binding site of the SARS-CoV-2 chimeric receptor-binding domain (RBD) with angiotensin-converting enzyme 2 (ACE2), while the FX potential binding displayed a higher binding energy than SX, but it could not bind at the RBD-ACE2 binding site [132]. Their inhibitor activity was confirmed with SARS-CoV-2 pseudovirus on HEK293 cells overexpressing ACE2, revealing a better effect of SX on the inhibition of viral infection. Although further studies are needed to elucidate the underlying mechanism, these results provide useful information on the application of these marine carotenoids for the treatment and prevention of COVID-19. Their possible application as a new therapeutic agent in the treatment and/or prevention of the severe inflammatory syndrome related to SARS-CoV-2 infection has been further supported by the in silico identification and in vitro validation of the binding between xanthophyll diatoxanthin (Dt) and ACE2 protein [133]. 

### 3.2. Bioactive Peptides’ Prediction and Validation

As previously stated, tuna peptides are known to have bioactive properties that are interesting for human health. Comprehensive studies of bioactive peptides from collagen by-products of the tuna fishery industry, especially from collagen of the skin of tuna bigeye (*Thunnus obesus*), are still limited. However, a recent work used an integrated method to search for bioactive peptides from the by-product of the tuna skin. A combination approach is considered cheap, easy and fast in the research for bioactive peptides. The analysis of the different skin tuna fractions using the BIOPEP website highlighted the presence of peptides with antidiabetic, antihypertensive and antioxidant properties [134]. The tool also allowed for the analysis of the peptide characteristics in terms of sequence length, molecular weight, isoelectric point, net charge and hydrophobicity. Bioactive peptides with antidiabetic, antihypertensive and antioxidant properties, and peptides with other bioactive properties, generally have the N terminal of a non-polar (hydrophobic) amino acid. This finding is in agreement with previous researchers, who also linked bioactive properties with hydrophobicity [86]. The bioactive peptides appeared as sequences of 2–6 amino acids with a molecular weight of 132.05–579.31. This range of amino acid residue lengths matches the range of bioactive peptide residue, as reported by previous investigators. The use of the DPPH assay validated the antioxidant activity of the skin tuna fractions.

Another interesting tool to predict peptides’ bioactivity is Peptide Ranker (PepRank) web-based application (http://bioware.ucd.ie/~compass/biowareweb/Server_pages/help/peptideranker/help.php; accessed on 25 January 2023). PepRank is an in silico tool that predicts the probability of a peptide being bioactive and that can rank peptide sets according to structure–function patterns [113]. A comprehensive study of peptidomic data of protein hydrolysates prepared from Atlantic Sea cucumber by-products predicted the probability of the bioactivity of each submitted peptide. The peptides were extracted from different sea cucumber body parts. Among all the predicted values, flowers and internal organs shared the same peptide sequence (GPPGPQWPLDF) for the highest predicted bioactivity. The predicted bioactive peptides were then analyzed for their specific bioactive potential, with an emphasis on antioxidant and ACE inhibitor activities, using the BIOPEP database. Similarly, BIOPEP and PepRank have been used to predict peptides’ bioactivity in in silico digestion products of carp collagen chains using papain and pepsin [135]. All of the antioxidative peptides virtually generated from the enzymes were dipeptides with free radical scavenging activity and oxygen radical scavenging activity, as predicted by the BIOPEP tool. Virtual digestion of carp collagen with papain theoretically released the highest number of antioxidant peptide sequences, and the dipeptide sequences were the most abundant. Virtual digestion of carp collagen with pepsin theoretically released the dipeptide and tripeptide antioxidative sequences. However, the virtual digestion of carp collagen with a mixture of pepsin and papain enzymes showed the maximum number of antioxidative peptides compared to each protease alone. The potential of the antioxidant peptides was calculated using the PepRank. The peptide generated from pepsin hydrolysis showed the maximum peptide ranking score. In vitro hydrolysis of the extracted carp collagens was carried out using papain and pepsin in two steps (the first step in pepsin at pH 2.0 and the second step in papain at pH 6.0), and DPPH radical scavenging activity of the collagen hydrolysates validated the predicted antioxidant activity. The utilization of fish processing by-products (skin, scale and swim bladder) for the extraction of collagen and their re-valorization is an eco-friendly waste management approach in food industries. These studies provided an additional basis for the development of carp collagen utilization as a precursor of antioxidant peptides to be used in nutraceutical and biomedical fields [135]. 

### 3.3. Identification of Marine Protein with Antioxidant Activity and Validation

Endogenous antioxidant enzymes, including superoxide dismutase, catalase, glutathione, thioredoxin and glutathione peroxidase, participate in different mechanisms that reduce ROS surpluses. Several in silico tools could be of benefit in the identification of antioxidant enzymes from marine organisms. These tools allow researchers to predict the amino acid sequence starting from the nucleic acid sequence, align sequences from several organisms to identify sequence similarities and conservation, predict the possible 2D and 3D structures with the identification of putative catalytic sites and finally, as we mentioned above, prdiect the possible protein interactors.

An interesting study identified a rock bream (*Oplegnathus fasciatus*) thioredoxin reductase 3-like molecule (RbTrxR-3) using in silico prediction [136]. The authors analyzed the homology and phylogenesis of RbTrxR-3, identifying it as a TrxR family ortholog. The in vitro and in vivo studies validated the importance of fish physiology, in particular, in pathogen invasion, along with functional properties that are important in host antioxidant defense. The transcriptional response of RbTrxR-3 in the livers of rock breams under live pathogen stress correlated with the temporal mRNA expression pattern of its putative substrate, RbTrx-1, suggesting that it may play an important role in immune or post-immune responses. In addition, RbTrxR-3 possesses a typical functional property of TrxR family members, suggesting their functional homology. Detectable thiol reductase activity of RbTrxR-3 further indicates that RbTrxR-3 plausibly plays a role in defending against oxidative stress [136]. 

A similar process was used to identify and characterize a copper–zinc superoxide dismutase (CuZnSOD) [137] from *Hippocampus abdominalis* (HaCuZnSOD). Starting from a cDNA sequence (accession number KU665493) from the seahorse cDNA database, the use of the basic local alignment search tool (BLAST) algorithm (http://blast.ncbi.nlm.nih.gov/Blast.cgi; accessed on 21 January 2023) allowed the identification of the HaCuZnSOD coding sequence and its corresponding protein sequence. In silico characterization of the putative protein sequence was carried out using ClustalW (http://www.ebi.ac.uk/Tools/msa/clustalw2/; accessed on 21 January 2023) and the neighbor-joining (NJ) method in MEGA (ver. 5.0) for the analysis of homology and evolutionary relationships of HaCuZnSOD with orthologs of other species. The protein structure was in silico characterized using the ExPASy PROSITE Database (http://prosite.expasy.org/; accessed on 21 January 2023) and Motif Scan (http://myhits.isb-sib.ch/cgi-bin/motif_scan; accessed on 21 January 2023), as well as I-TASSER (http://zhanglab.ccmb.med.umich.edu/I-TASSER/; accessed on 21 January 2023) and SWISS-MODEL (http://swissmodel.expasy.org/; accessed on 21 January 2023) for the tertiary structure prediction. Recombinant HaCuZnSOD (rHaCuZnSOD) was overexpressed in a bacterial system and purified for further characterization and validation. In particular, the antioxidant activity of rHaCuZnSOD was investigated using the conventional xanthine/xanthine oxidase (xanthine/XOD) assay. Significantly higher activities were found for metal-supplemented rHaCuZnSOD compared to non-supplementation. Moreover, the peroxidation function of rHaCuZnSOD was assessed by investigating cell viability using a 3-(4,5-dimethylthiazol-2-yl)-2,5-diphenyl-2H-tetrazolium bromide (MTT) assay on THP-1 human cells under cytotoxic conditions after H_2_O_2_ treatment. Cell viability increased in the presence of rHaCuZnSOD in a dose-dependent manner. The effect included a decrease in intracellular ROS measured by flow cytometry. All these data demonstrate that the use of in silico tools can identify and predict several antioxidant characteristics of a protein, as have been successfully validated through in vitro and in vivo experiments (Table 2) [137].

## 4. Conclusions and Future Perspectives

Marine organisms have been shown to be excellent sources of natural products with antioxidant activities, including scavenger molecules and enzymes. The most common pipeline followed by researchers in order to discover new antioxidants starts generally with the sampling of the marine organism, its culturing (when possible), chemical extraction/fractionation and assay testing for a specific activity. Once the activity is found, additional analyses are performed to identify the compound/s of interest and characterize its mechanism of action. The increasing number of -omics resources, the advent of new sequencing technologies, the lowering of the sequencing prices and the new or improved bioinformatic tools have allowed the development of in silico searches for new marine natural products and new bioactivities, as well as descriptions/predictions of molecule interactions with putative targets for possible human applications, especially in the pharmaceutical, nutraceutical and cosmeceutical fields. Recently, various papers have reported docking tools, compared them and tested their efficacy in virtual screening against a wide range of protein targets [122,139,140,141,142]. Very recently, these in silico studies have begun to focus on marine organisms and marine natural products [143]. This approach requires low costs and offers the possibility of using an ecofriendly research strategy (no need to sample several species and induce damage to the environment). More than one tool is often used for the same experiment, such as a tool for studying a transcript sequence, one for translating it into a protein sequence and one for predicting the tertiary structure and/or for studying the active site or modeling with a possible substrate. However, this in silico identification should be followed by validation steps to confirm the presence of the compound and characterize its activity. Production or synthesis of the compound/enzyme of interest will be necessary for the validation step, and this may be not an easy task in terms of species culturing (not always possible), heterologous expression and chemical synthesis. We need further studies and technology improvements to bring this along (e.g., for cyclo-peptides and complex polyketides). Our review highlights that these kinds of studies are still few in number but have been increasing in recent years. We expect that the number of in silico studies will further increase and make a great contribution to the discovery of new antioxidant products or better characterization of known ones, to keep pace with the increasing industrial demand.

## Figures and Tables

**Table 1:** Representative molecular in silico prediction programs, their main characteristics, free availability and corresponding references.

Tools	Information	Availability	References
Docking			
AutoDock4	Grid-based flexible docking prediction	Free	[89]
AutoDock Vina	Grid-based flexible docking prediction using multithreading	Free	[90]
Flexible CDOCKER	Docking prediction exploring the conformational space simultaneously of ligands and protein configurations	Free only for academic, government and nonprofit labs	[91]
FireDock	Rescoring and refinement of docking solutions	Free	[92]
PatchDock	Predicts protein–protein and protein–small molecule docking	Free	[93]
Dockey	Analysis of non-covalent interactions between small molecules and proteins, performing cross-docking with multiple receptors and ligands	Free	[94]
iMOLSDOCK	Induced-fit docking algorithm, with improvement of the receptor flexibility	Free	[95]
Bioactivity			
BIOPEP-UWM	Database of peptides, proteins, amino acids and allergens	Free	[96,97]
PepRank	Predicts bioactivity of a peptide	Free	[98]
AllergenFP	Predicts allergenicity of a peptide	Free	[99]
ToxinPred	Predicts toxicity of a peptide	Free	[100]
Protein structure			
ExPASy	Database of resources from the Swiss Institute of Bionformatics (SIB)	Free	[101]
SWISS-MODEL Repository	Generates models based on homology modeling	Free	[102]
I-TASSER	Predicts 3D protein structures	Free	[103]
Pfeature	Predicts protein residue-level annotation, protein function and chemically modified peptides’ function	Free	[104]
AlphaFold	Deep learning algorithm that predicts protein structure, even if there is not a similar one known	Free	[105]
Pharmacophore			
Phase	Pharmacophore modeling with tree-based partitioning algorithm	Free	[106]
MOE	Pharmacophore modeling, in which for the 3D pharmacophore database, a consensus query can be used from several aligned molecules	Commercial	[107]
LigandScout	Pharmacophore modeling, which also allows researchers to compare the common binding modes of pharmacophores and molecules	Commercial	[108]

**Table 2:** Representative examples of in silico analysis and validation to discover antioxidant properties of marine origins.

Organism	Antioxidant Compound/Enzyme	In Silico Prediction Tool	Validation Assay	Possible Application Field	Reference
Alga *Caulerpa racemosa*	Crude polyphenolic extract (CPE), caulerpin	AutoDock	DPPH (2, 2-diphenyl-1-picryl-hydrazylhydrate) radical photometric assay	Diabetic conditions, breast cancer	[119]
Bacterium *Pseudomonas aeruginosa*	Hexane ethyl acetate (HPAEtOAcE) fraction, 5-(1H-indol-3-yl)-4-pentyl-1,3-oxazole-2-carboxylic acid (Compound 1)	CDOCKER	DPPH (2, 2-diphenyl-1-picryl-hydrazylhydrate) radical photometric assay	Drug against several harmful pathogens, methicillin-resistant Staphylococcus aureus (MRSA)	[120]
Bacterium *Streptomyces mangrovisoli*	Cyclo (L-Leucyl-L-Prolyl) peptide/CLP	PatchDock	Co-immunoprecipitation	Triple negative breast cancer (TNBC)	[131]
Red Sea sponge *Diacarnus erythraeanus*	(−)-Muqubilin (Muq)	AutoDock Vina (version 1.1.2)	Luciferase assay to validate the agonistic effect	Neurological diseases	[121]
Tuna fish	KEFT, EEASA and RYDD peptides	CDOCKER	In vivo administration and evaluation of protein and transcript levels of antioxidant enzymes	Keap1/Nrf2/ARE antioxidant pathway regulation	[138]
Seaweeds and diatoms	Fucoxanthin (FX), siphonaxanthin (SX), diatoxanthin (Dt)	AutoDock Vina (version 1.1.2)	In vitro simulation of viral infection	Treatment and/or prevention of severe inflammatory syndrome	[132,133]
Tuna bigeye (*Thunnus obesus*)	Bioactive peptides	BIOPEP	DPPH (2, 2-diphenyl-1-picryl-hydrazylhydrate) radical photometric assay	Food and health applications	[134]
Atlantic sea cucumber	Peptide sequence (GPPGPQWPLDF)	BIOPEP and PepRank	DPPH (2, 2-diphenyl-1-picryl-hydrazylhydrate) radical photometric assay	Food industries	[135]
Rock bream (*Oplegnathus fasciatus*)	RbTrxR-3	EMBOSS Needle and ClustalW; ExPASy PROSITE; SECISearch; ExPASy ProtParam tool	Thiol-reductase activity	Response to pathogen stress	[136]
*Hippocampus abdominalis*	HaCuZnSOD	ClustalW; ExPASy PROSITE; Motif Scan; I-TASSER; SWISS-MODEL	Xanthine/xanthine oxidase (xanthine/XOD) assay	Host antioxidant defense	[137]

## Data Availability

Not applicable.
